# Microenterprise intervention to reduce sexual risk behaviors and increase employment and HIV preventive practices in economically-vulnerable African-American young adults (EMERGE): protocol for a feasibility randomized clinical trial

**DOI:** 10.1186/s13063-019-3529-7

**Published:** 2019-07-17

**Authors:** Larissa Jennings Mayo-Wilson, Nancy E. Glass, Fred M. Ssewamala, Sebastian Linnemayr, Jessica Coleman, Fatmata Timbo, Matthew W. Johnson, Melissa Davoust, Alain Labrique, Gayane Yenokyan, Brian Dodge, Carl Latkin

**Affiliations:** 1 Department of International Health, Social and Behavioral Interventions Program, Johns Hopkins Bloomberg School of Public Health, 615 N. Wolfe Street, Room E5038, Baltimore, MD 21205 USA; 2 Department of Applied Health Science, Indiana University School of Public Health, 1025 E. 7th Street, Bloomington, IN USA; 3 Johns Hopkins University School of Nursing, 525 N. Wolfe Street, Baltimore, MD USA; 4 Washington University in St. Louis, The Brown School, Goldfarb, One Brookings, Drive, St. Louis, MO USA; 5 RAND Corporation, 1776 Main Street, Santa Monica, CA USA; 6 Behavioral Pharmacology Research, Johns Hopkins University School of Medicine, 5510 Nathan Shock Drive, Baltimore, MD USA; 7 Department of Biostatistics, Johns Hopkins Bloomberg School of Public Health, 615 N. Wolfe Street, Baltimore, MD USA; 8 Department of Health, Behavior and Society, Johns Hopkins Bloomberg School of Public Health, 624 N Broadway, Hampton House 737, Baltimore, MD USA

**Keywords:** HIV, Sexual risk behaviors, Homeless, Text messages, Young adults, Baltimore, Economic, Unemployment, Feasibility, Clinical trial

## Abstract

**Background:**

Economic vulnerability, such as homelessness and unemployment, contributes to the HIV risk among racial minorities in the U.S., who are disproportionately infected. Yet, few economic-strengthening interventions have been adapted for HIV prevention in economically-vulnerable African-American young adults. Engaging Microenterprise for Resource Generation and Health Empowerment (EMERGE) is a feasibility randomized clinical trial of an HIV prevention microenterprise intervention with integrated text messages (“nudges”) that are informed by behavioral economic principles. The trial aims to reduce sexual risk behaviors and increase employment and uptake of HIV preventive behaviors.

**Methods/design:**

In total, 40 young adults who are African-American, aged 18–24, live in Baltimore City, have experienced at least one episode of homelessness in the last 12 months, are unemployed or underemployed (fewer than 10 h per week), are not enrolled in school, own a cell phone with text messaging, and report at least one episode of unprotected or unsafe sex in the prior 12 months will be recruited from two community-based organizations providing residential supportive services to urban youth. Participants will undergo a 3-week run-in period and thereafter be randomly assigned to one of two groups with active interventions for 20 weeks. The first group (“comparison”) will receive text messages with information on job openings. The second group (“experimental”) will receive text messages with information on job openings plus information on HIV prevention and business educational sessions, a mentored apprenticeship, and a start-up grant, and business and HIV prevention text messages based on principles from behavioral economics. The two primary outcomes relate to the feasibility of conducting a larger trial. Secondary outcomes relate to employment, sexual risk behaviors, and HIV preventive practices. All participants will be assessed using an in-person questionnaire at pre-intervention (prior to randomization) and at 3 weeks post-intervention. To obtain repeated, longitudinal measures, participants will be assessed weekly using text message surveys from pre-intervention up to 3 weeks post-intervention.

**Discussion:**

This study will be one of the first U.S.-based feasibility randomized clinical trials of an HIV prevention microenterprise intervention for economically-vulnerable African-American young adults. The findings will inform whether and how to conduct a larger efficacy trial for HIV risk reduction in this population.

**Trial registration:**

ClinicalTrials.gov, NCT03766165. Registered on 4 December 2018.

**Electronic supplementary material:**

The online version of this article (10.1186/s13063-019-3529-7) contains supplementary material, which is available to authorized users.

## Background

African-American young adults are disproportionately affected by the HIV epidemic. Although representing only 12% of the United States population [1], African-Americans make up nearly half (42%) of all U.S. HIV infections [2]. Altogether, 61% and 34% of HIV infections in African-Americans are attributed to male-to-male sexual contact or heterosexual contact, respectively [2]. The rate of new HIV infections is 8.3 times higher in African-Americans compared to non-Hispanic whites [2]. Most U.S. HIV infections are concentrated in urban metropolitan areas [3]. This study is conducted in Baltimore, Maryland. According to the Centers for Disease Control and Prevention, Baltimore is ranked 23rd in the number of persons living with diagnosed HIV infection (prevalence) in 2017 out of 108 metropolitan areas [3]. In total, 82% of adult and adolescent HIV diagnoses in Baltimore were in non-Hispanic Blacks (African-American) [4]. In addition, young adults in Baltimore, MD, aged 20–29, make up the largest proportion of HIV diagnoses (29%) compared to any other age group [4]. Yet, despite persistent racial disparities in HIV infection, there have been few intervention advances to address the epidemic in predominately African-American settings.

According to UNAIDS, the U.S. has a concentrated HIV epidemic that has greatly affected impoverished urban areas [5, 6]. Data from the U.S. National HIV Behavioral Surveillance System has shown that economic vulnerability, in the form of low income, unemployment, and homelessness, is associated with increased HIV risk. Controlling for factors often associated with HIV (i.e., male-to-male sexual contact and injected drug use), the HIV prevalence rate is 2.1 times higher among individuals with income at or below the U.S. poverty threshold compared to those above [5, 6], and 2.6 times higher among unemployed individuals compared to those in employment [5, 6]. Homelessness in the past year is also associated with 1.8 times higher HIV prevalence [5, 6]. Low education and low annual household income (≤$9999) are also significantly associated with higher HIV prevalence [5, 6]. Furthermore, HIV prevalence in U.S. urban poverty areas is alarmingly high at 2.1%, over seven times the HIV prevalence in the U.S. (0.3%) [1, 4, 5].

Young adults make up an increasing proportion of the urban homeless and unemployed population. Limited economic resources can create a short-term imperative among economically-vulnerable youth to engage in high-risk income-earning activities that contribute to HIV risk, such as transactional sex or engagement in theft or the illegal drug economy, which are linked to the adverse consequences associated with HIV risk (i.e., substance abuse, incarceration, and intimate partner violence) [7–9]. Limited economic resources may also lead to a loss of hope and agency that diminish motivations to avoid exposure to future HIV infection [7–9].

It has been shown in low-income countries that microenterprises (or very small businesses) can improve sexual attitudes [10, 11], sexual risk behaviors [12–17], and HIV communication and testing [13, 15], by combining HIV and microbusiness training, mentoring, and small grants [18]. According to asset theory, increases in assets can influence individual health behaviors by motivating protective attitudes to avoid negative consequences [19–21]. Yet, the absence of published studies on HIV-prevention microenterprise interventions for economically-vulnerable African-Americans has hindered efforts to reduce HIV risk in this population. Prior research has also not examined the potential synergies of integrating text messaging and the principles of behavioral economics into HIV prevention microenterprise interventions. We will explore the use of low-cost text messages to conduct outcome assessments. Text messages on healthy business and sexual practices will also be used in the form of small nudges, as suggested by behavioral economics, to motivate behavior change [22]. To our knowledge, this study will be among the first U.S.-based feasibility randomized clinical trials of an HIV prevention microenterprise intervention for economically-vulnerable African-American young adults. The findings will inform whether and how to conduct a larger efficacy trial for HIV risk reduction in this population.

## Methods/design

### Aims

#### Primary aim

The primary aim is to examine the feasibility of conducting a larger trial of an HIV prevention microenterprise intervention for economically-vulnerable African-American young adults.

#### Secondary exploratory aims

To explore the effects of an HIV prevention microenterprise intervention on:

1. sexual risk behaviors in economically-vulnerable African-American young adults
2. employment of economically-vulnerable African-American young adults
3. HIV preventive behaviors in economically-vulnerable African-American young adults.

### Study design

This feasibility trial is a two-group parallel design with a 1:1 allocation ratio.

### Study registration

**Fig. 1:**
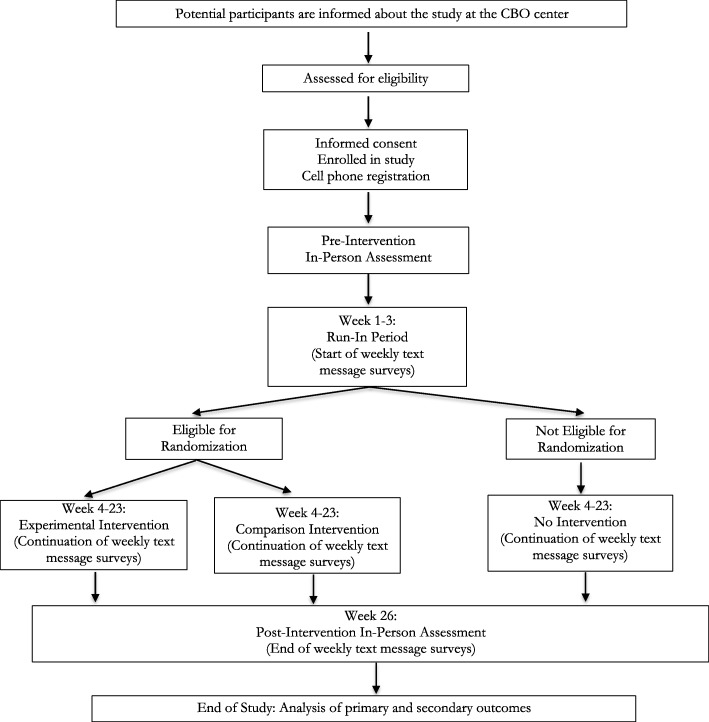
Participant flow diagram. CBO Community-based organization

**Fig. 2:**
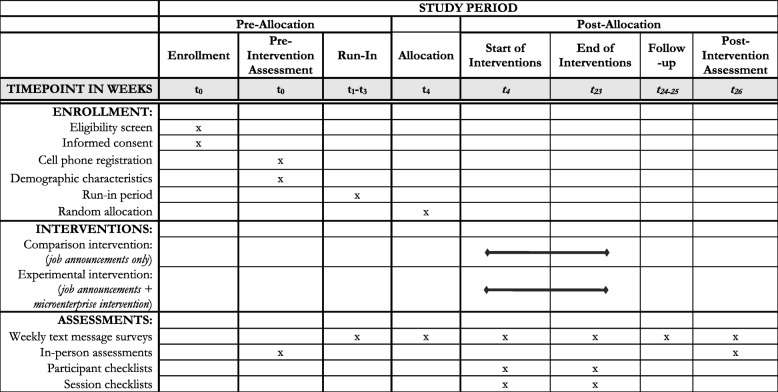
Schedule of enrollment, interventions, and assessments according to the Standard Protocol Items: Recommendations for Intervention Trials (SPIRIT) Diagram

The trial is registered at ClinicalTrials.gov (NCT03766165). It is entitled the EMERGE Project, for Engaging Microenterprise for Resource Generation and Health Empowerment (K01MH107310). LJMW is the principal investigator (PI) and will oversee the trial. JC and FT are the senior research coordinators (SRCs) and will manage the implementation of the trial. This protocol manuscript has been prepared according to the Standard Protocol Items: Recommendations for Interventional Trials (SPIRIT) Statement [23] and will be reported according to the Consolidated Standards of Reporting Trials Statement for Social and Psychological Interventions (CONSORT-SPI) [24] and the extension for randomized pilot and feasibility trials [25]. Figure 1 provides a participant flow diagram. A SPIRIT diagram for enrollment, interventions, and assessments is shown in Fig. 2. The SPIRIT 2013 checklist is provided as Additional file 1. The feasibility trial is active and ongoing. These methods are based on the EMERGE trial protocol (version 5 dated 23 January 2019), which has been approved by the institutional review board of Johns Hopkins Bloomberg School of Public Health (00008833). Any amendments made to the protocol will be reported on ClinicalTrials.gov and to the board.

### Setting

The feasibility trial will take place in Baltimore, MD, at two community-based organizations (CBOs), AIDS Interfaith Residential Services and Youth Opportunities! Baltimore. The CBOs provide transitional and emergency housing for young adults aged 18–24 who are experiencing homelessness. Each CBO has a resource center with certified counseling and social services, individual and group meeting rooms, staff offices, a computer lab, and a pamphlet booth with posted and printed resources on health and well-being. Participants will be recruited from and receive the interventions at the CBOs.

### Timeline

Recruitment occured from December 2018 to February 2019. The run-in period was conducted in February 2019. All eligible participants were randomized immediately after they have completed the run-in period. The interventions are expected to be conducted from March to July 2019.

Data will be collected for 26 weeks (weeks 1 to 26), which is equivalent to approximately 6 months. Participants will undergo an in-person pre-intervention assessment at the time of enrollment and be randomized in week 4 if they successfully complete a 3-week run-in period (weeks 1 to 3). Both groups will receive the assigned interventions concurrently for 20 weeks (weeks 4 to 23). An in-person post-intervention assessment will be conducted in week 26, equivalent to 3 weeks after the end of the interventions. For a subset of outcomes, participants will also complete a weekly text message survey from weeks 1 to 26.

### Eligibility criteria

Study eligibility will be determined using a screening tool during the in-person enrollment. Participants will be eligible to participate if they are African-American, aged 18–24, live in Baltimore, MD, have experienced at least one episode of homelessness in the last 12 months, are unemployed or employed for fewer than 10 h per week, are not enrolled in school, own a cell phone with text messaging function, and report at least one episode of unprotected or unsafe sex in the prior 12 months. Participants may enroll in the study regardless of gender identity or sexual orientation. Participants will be ineligible to participate if they are unwilling or unable to provide consent to participate.

### Sample size

A power calculation to estimate the sample size is not appropriate for a feasibility trial because the aim of the trial is not to establish efficacy [26]. Instead we determined that a minimum sample of 30 participants (15 in each group) would generate sufficient data to assess feasibility (defined as outcomes 1 to 7 in the outcomes section below) and to assess the acceptability of the assigned interventions, including rates of recruitment and retention in the trial. This was determined with reference to good practice recommendations for feasibility studies, which recommend sample sizes of between 24 and 50 [26–29] and as used in published study protocols of other feasibility randomized clinical trials [30–33]. The minimum sample size of 30 was inflated to 40 to allow for dropouts during the run-in period (15%) and dropouts following randomization (10%), which was estimated to be a total of 25% based on similar studies [34–37]. In the absence of any dropouts during the run-in period, we will randomize 40 participants (20 in each group). Assuming a feasibility of at least 70%, a sample size of 30 to 40 will allow us to estimate this proportion with a 15% point margin of error using a 95% confidence interval. We expect to screen approximately 60 participants to achieve the planned enrollment of 40.

### Recruitment

Potential participants will be recruited on-site at the two participating CBOs. A one-page recruitment flyer will be posted in the main building of each CBO. Designated CBO staff will inform potential participants of the study team’s scheduled visit days. One or two research assistants will be present on each scheduled visit day. CBO staff will be asked not to selectively flag or exclude potential participants for the study. On these visit days, the PI and/or a trained research assistant will introduce the study to a group of potential participants, who will have an opportunity to ask questions about the study and add their names to a sign-up sheet if they are interested and consider themselves to be potentially eligible to participate. On the same day, in order of sign-up, the PI or the trained research assistant will accompany each potential participant to a private room to complete the screening tool, administer informed consent, register the participant’s cell phone to the study’s online text messaging service (TextIt.in), and conduct the pre-intervention assessment. Written informed consent will include consent inclusive of the following: study enrollment, cell phone registration for receiving study text messages, conduct of study assessments, randomization to comparison or experimental intervention, use of data for evaluation, and publication of results.

To register participants’ cell phones to the online text messaging service, they will be invited to text “join” to the study phone number. Each participant will then undergo a brief in-person orientation regarding the weekly text message survey’s content, timing, and payment structure. Participants will be paid $15 in cash for completing the in-person pre-intervention assessment and an additional $15 in cash for completing the in-person post-intervention assessment. Participants will be paid $3 in cash each week for responding to each weekly text message survey (for a total of $78 from weeks 1 to 26). In the presence of the PI or a trained research assistant, the participant will also complete on their cell phone a mock, but otherwise identical version of the weekly text message survey to be used in the study. This will be done to confirm they have sufficient literacy and can read the text message questions and to clarify any points of confusion with the participant. The text survey is the only survey that the participants will read alone. All in-person assessments will be orally administered by the PI or a trained research assistant. The PI or a trained research assistant will also advise participants that they can opt out of the text message survey by texting “leave” at any time. The PI or a trained research assistant will also advise participants about how to increase privacy during the study period, such as activating cell phone passwords, deleting previously received text message surveys, sending survey responses only to the study’s phone number, and answering in a quiet and private space.

Several strategies will be used to achieve adequate participant enrollment. These include: asking CBO staff about the best times to visit the site, passing out recruitment flyers to interested participants, providing snacks and beverages for participants who are waiting to be screened and enrolled, and returning to the study site at least twice to recruit potential participants who were unavailable during previous visit days.

### Run-in period

Following the recruitment period, participants will begin a 3-week run-in period prior to randomization. The run-in period will be used to minimize dropouts after randomization by identifying participants who are likely to take up the intervention and to complete outcome assessments. It will exclude participants who are unlikely to take up activities that are like those used in the trial [34–39]. Participants who complete all three of the study’s run-in requirements will be eligible for randomization. These run-in requirements are: (1) to respond to three consecutive weekly text message surveys (weeks 1 to 3), (2) to email a brief statement to the study’s email address describing the type of microenterprise they would like to start by the end of week 2 (or to submit a handwritten statement), and (3) to attend a 30-min group meeting in week 3 that will be held at the same time and place as the proposed educational sessions in weeks 4 to 23. Invitations to the group meeting in week 3 will be conditional on successful completion of the first two run-in requirements. Participants will receive text message reminders to complete the run-in requirements.

The run-in requirements were chosen to reflect components of the study’s assessment activities and experimental intervention, which include responding to weekly assessments, attendance at in-person educational sessions, and participation in non-meeting intervention activities related to intervention exposure (i.e., a mentored apprenticeship). Participants will be informed of the run-in requirements for randomization during the process of informed consent. Participants who do not successfully complete all run-in requirements and are, therefore, ineligible for randomization may choose either to: (1) withdraw from the study or (2) remain in the study and complete only the study assessments (i.e., weekly text message surveys and in-person post-intervention assessment). Participants who are not randomized but chose to continue completing the weekly text message surveys and to complete the in-person post-intervention assessment will be paid the same amount as randomized participants for completing these assessments. Information on run-in failures will be documented to inform the study’s examination of recruitment and retention.

### Allocation

The PI or a trained research assistant will enroll participants prior to the run-in period and prior to the assignment to the comparison or experimental intervention. A biostatistician who is not involved in recruitment, intervention implementation, or outcome assessment will use a computer to generate the allocation sequence and assign all participants, at the same time, to the comparison or experimental intervention. Participants will be randomized in a 1:1 ratio, stratified by CBO to allow equal numbers of CBO participants in each study group.

### Experimental intervention

The experimental intervention will be delivered over 20 weeks. The goal is to reduce sexual risk behaviors and increase employment and uptake of HIV preventive behaviors by building skills and through motivational messaging and financial support. It is based on prior qualitative formative research conducted by the PI that examined health-focused entrepreneurial education [40, 41], cell phone behaviors [42], uptake of text message surveys, and behavioral economics relating to sexual risk behaviors [43] in young adults experiencing homelessness. It is also based on prior published microenterprise interventions for HIV risk reduction used primarily in low-income countries, and adapted for use in a U.S. urban minority setting [13, 44–52]. Participants assigned to the experimental intervention will receive the following:

#### One text message each week on job openings

We will use TextIt.in to deliver an automated text message every Monday with information on one job opening in Baltimore appropriate for a young adult at or slightly above or below high school diploma or equivalent training. All participants will receive the same job announcement text message. The announcements will not be individualized.

#### One 3-hour educational session each week on starting a business or on HIV prevention

The PI and/or one or both SRCs will lead a classroom-based educational group session at the CBO resource center. The sessions will be held on Wednesdays. Snacks will be provided. Each session will include a 45-min PowerPoint presentation, group discussions, small-group activities, and completion of the session and participant checklists. Participants will also receive related handouts and forms. Table 1 lists the topics of the educational sessions. The sessions will aim:

1. to improve knowledge of planning, initiating, and managing an income-generating microenterprise;
2. to improve entrepreneurial skills and experiences that can enhance employability;
3. to improve knowledge of the associations between economic vulnerability and HIV risk in U.S. racial minority young adults;
4. to improve knowledge of sexual risk behaviors (such as unprotected sex, sex while high or drunk, sex with persons with unknown HIV status, etc.) and ways to minimize them in exchange for safer sex behaviors;
5. to improve knowledge and uptake of HIV-prevention practices (such as condom use, discussions with sexual partners, HIV testing, use of pre-exposure prophylaxis HIV medications, and safer sex behaviors).

**Table 1:** List of topics for weekly educational sessions in the experimental intervention

Session no.	Session topic
1	Introduction to the EMERGE Project
2	Introduction to Entrepreneurship
3	Developing a Microbusiness Idea
4	Developing a Microbusiness Plan and Budget
5	Poverty and HIV in Baltimore City (HIV 1)
6	Safer Sex Communication and Practices (HIV 2)
7	Working with a Business Mentor
8	Microbusiness Registration and Launch
9	Managing Personal and Business Finances
10	Behavioral Economics and Sexual Risk-Taking (HIV 3)
11	Using Your Money to Prevent HIV (HIV 4)
12	Expanding Your Microbusiness
13	Marketing and Managing Microbusiness Risks
14	Acquiring New Skills for Your Microbusiness
15	Accessing HIV Prevention Technologies (HIV 5)
16	Avoiding Costs of Unsafe Sex (HIV 6)
17	Real Baltimore Business Owners: Questions Answered
18	Group Presentations and Feedback
19	Preparing for Small Business Taxes
20	Closing and Summary

In addition to microenterprise education, to address the economic drivers of HIV risk, the sessions will also integrate principles from asset theory, which suggests that individuals are more likely to engage in positive behaviors perceived as protecting current or future assets [19–21]. Specifically, the sessions will assist participants:

(6) to identify financial and sexual health goals (such as earning an income or discussing safer sex with sexual partners);
(7) to identify financial and sexual health assets to protect themselves from negative consequences (such as maintaining positive relationships with microbusiness customers or accompanying a new sexual partner to HIV prevention and care services).

The sessions will also integrate principles from behavioral economics, which suggests that individuals make health-related decisions based on their costs and benefits, but are also influenced by key behavioral economic biases relating to delays in time, subjective value, and incomplete information [53–58]. Specifically, the sessions will aim:

(8) to improve awareness of behavioral economics biases relating to sexual risk behaviors (such as preferring the immediate gratification of condomless sex);
(9) to improve awareness of the short-term benefits of safer sex behaviors and HIV preventive practices (such as being informed, being safe, or having peace of mind);
(10) to develop a financial plan for HIV risk reduction (such as saving money for buying condoms or traveling to HIV prevention clinics).

Ultimately, the educational sessions will aim to encourage safe practices that reduce the risk of infection to sexual partners who are HIV-negative and to reduce co-infections with existing HIV-positive partners.

#### One mentored apprenticeship during the intervention period

The PI and SRCs will recruit approximately four to six mentors to participate in the study. Eligible mentors will be aged 25 and older, live in Baltimore, own a small business in Baltimore, and speak English. The mentors will attend an orientation session led by the PI and/or SRCs prior to participation. Each mentor will receive an honorarium of $100. They will also receive a brief summary of their matched mentees and their microbusiness interests. We will introduce participants to these mentors in weeks 7 to 10. Mentors will be expected to attend a minimum of three educational sessions during which they will talk about their business experience and provide feedback to the mentees on their microbusiness goals. Mentors will also provide advice to their mentees by text, phone, online, or in-person over the 20 weeks of the intervention. Each mentor will be expected to hire (or connect for hire) mentees for a minimum of 24 h over a 3-week period (approximately 8 hours per week) at or above the State of Maryland minimum wage.

#### One microbusiness start-up grant

Each participant will receive a start-up grant (repayment not required) for $11000 paid by check in six payments of $100, $200, $200, $200, $200, and $200 U.S. dollars. The grant will be used for purchasing microbusiness supplies, marketing, communication, and travel (if required to sell goods and services). Participants must meet all the required milestones to receive each of the six payments. These milestones will include: (1) attendance at educational sessions, (2) completion of weekly text message surveys, (3) development of a microbusiness budget that lists the expected use of funds, and (4) where applicable, submission of receipts relevant for microbusiness purchases. For example, participants may be asked to show other evidence of a business such as business cards, a website, or a photo of a product or service transaction. We will determine whilst the intervention is underway when to make payments to participants depending on their progress.

#### Three text messages each week on running a microenterprise or HIV prevention

We will use TextIt.in to deliver three automated text messages each week with information on running a microenterprise and HIV prevention. One text message will be delivered on Tuesday, Wednesday, and Thursday, respectively. The HIV prevention and microenterprise weekly text messages (nudges) will reiterate key messages from the educational sessions described above.

### Comparison intervention

The comparison intervention will be conducted concurrently with the experimental intervention. Participants assigned to the comparison intervention will receive one text message each week on job openings. The job announcements sent each Monday to participants assigned to the experimental intervention will also be sent each Monday to participants assigned to the comparison intervention.

The comparison intervention was selected to promote study retention. Formative research conducted by the PI found that potential participants value job announcements and payments for completing text message surveys. Therefore, the goal of the comparison intervention is to reduce potential non-participation among participants randomly assigned to a study group that might otherwise be considered to have little value. The comparison intervention may also create similar benefit expectancies and non-specific factors such as weekly contact with the investigators. Payments for responding to the weekly text survey are the only financial incentive that participants in the comparison group will receive.

### Masking

A fully masked design is not possible because participants will know to which intervention group they have been assigned, and the study team members administering the microenterprise intervention will know participants’ assignments. However, as both interventions are economic-strengthening activities, the similarities of the interventions may reduce possible biases in the expectations of benefits by participants. Both interventions will be described as novel activities aiming to improve employment for young adults in Baltimore. To reduce contamination, participants assigned to the experimental intervention will be asked to refrain from talking about the intervention to peers assigned to the comparison intervention.

To mask the investigators, the following strategies will be used: (1) a research assistant who is not involved in delivering the interventions will conduct the in-person post-intervention assessment, (2) participants will be asked not to disclose their group assignment to the research assistant, and (3) the PI will provide a masked dataset to a research analyst so that statistical analyses can be performed without them knowing the group assignments. Feasibility outcome data obtained only for the experimental intervention group using session and participant checklists cannot be masked and will be assessed and analyzed by the PI, SRCs, and/or an unmasked research assistant.

### Retention

To promote retention and completion of follow-ups, enrolled participants will be asked to provide a personal email address, their cell phone number, and the name and phone number of one adult contact. This information will be stored on a locator form accessible only to the research team. If a participant temporarily ceases interacting with the study, a research assistant will then call, text, or email the participant, call the adult contact, or inquire among staff at the CBO where the participant was recruited. In addition, all participants will be reminded of the post-intervention assessment by text message. Participants have the right to withdraw from the study at any time. If a participant withdraws, the study will not collect additional study information from them after the time of withdrawal. Previously collected data will still be used in the analyses unless the participant withdraws consent. Where possible, the reason for participant withdrawal will be collected.

### Outcomes

The primary and secondary outcomes of the study are described below. The two primary outcomes for feasibility are:

1. Proportion of participants in both groups who responded to 70% or more of the weekly text message surveys (measured in week 26): We will use downloaded response data from the online text messaging service (TextIt.in) to tabulate the number and proportion of participants in both groups responding each week to the weekly text message survey. A participant will be categorized as responding to the survey if they provide a valid response to one or more text message questions, such as “yes” or “no,” a numerical response, a free-form text answer (i.e., “condoms” or “abstinence”), or a “skip” response to proceed to the next question. Non-responders will be defined as enrolled participants who did not return a valid text message response to any of the text message questions in a given week. At the end of the study period (week 26), we will calculate the number and proportion of participants who responded to 70% or more of the weekly text message surveys.
2. Proportion of experimental intervention participants who completed 70% or more of the intervention activities (measured in week 23): For participants assigned to the experimental intervention, the PI or an SRC will administer in-person a structured weekly participant checklist at the end of each educational session. This will assess session attendance and ask if the participant received one or more informational text messages in the prior week, if the participant received one or more mentor contacts (i.e., in-person, email, text, online, or by phone) in the prior week, and if the participant spent one or more grant payments in the prior week. At the end of the intervention period (week 23), we will then calculate the number and proportion of participants assigned to the experimental intervention who completed each activity (i.e., text message receipt, session attendance, grant spending, and mentor contact) in a given week and completed the full experimental intervention (70% or more of the intervention activities).

Primary outcome 1 assesses the feasibility of the text message survey for repeated, longitudinal data collection of outcomes in a potential larger trial. Primary outcome 2 evaluates the feasibility of achieving adequate participant participation over time in the experimental intervention to inform whether and how to modify the experimental intervention and the study protocol in a potential larger trial.

The secondary outcomes are:

3. Proportion of all participants who received one or more informational text messages (measured weekly in weeks 4 to 23): We will use downloaded data from the online text messaging service (TextIt.in) to tabulate the number and proportion of participants who were sent one or more text messages each week without an error message. In addition, the PI or an SRC will administer in-person a structured weekly participant checklist at the end of each educational session (for the experimental intervention group only) that will document whether a participant received one or more informational text messages.
4. Proportion of all participants who responded to the text message survey (measured weekly in weeks 1 to 26): We will use downloaded response data from the online text messaging service (TextIt.in) to tabulate the number and proportion of participants in both groups responding each week to the weekly text message survey. This is a weekly outcome that will be used to tabulate primary outcome 1.
5. Proportion of experimental intervention participants who attended an educational session (measured weekly in weeks 4 to 23): The structured weekly participant checklist will document the attendance of the participant the in-person educational session at the CBO center.
6. Proportion of experimental intervention participants who received one or more mentor contacts (measured weekly in weeks 4 to 23): The structured weekly participant checklist will document whether each participant has corresponded with their microbusiness mentor in-person, by email, by text, online, or via a phone call.
7. Proportion of experimental intervention participants who spent one or more grant payments (measured weekly in weeks 4 to 23): The structured weekly participant checklist will document whether each participant has spent one or more of the grant payments.
8. Proportion of participants in each group who reported engaging in one or more unprotected sex acts in the last week (measured weekly in weeks 1 to 26): During the weekly text message survey, participants will be asked about unprotected sex acts: “In the last 7 days, how many times did you have any type of sex without a condom and without any HIV medications?”
9. Proportion of participants in each group who reported engaging in one or more unprotected or unsafe sex acts in the last month (measured in weeks 1 and 26): During the in-person pre-intervention and post-intervention assessments, participants will be asked a list of binary questions about unprotected and unsafe sex acts: “In the last 30 days, have you had vaginal or anal sex without a condom? Without taking HIV prevention medications? While drunk? While high? With someone whose HIV status you did not know? With concurrent partners? With a stranger in exchange for food, housing, money, drugs, or medications?”
10. Proportion of participants in each group who reported engaging in one or more safer sex acts in the last week (measured weekly in weeks 1 to 26): During the weekly text message survey, participants will be asked about safer sex acts: “In the last 7 days, how many times did you have oral sex only? Sex while sober? Sex while using a lubricant?”
11. Proportion of participants in each group who reported engaging in one or more safer sex acts in the last month (measured in weeks 1 and 26): During the in-person pre-intervention and post-intervention assessments, participants will be asked a list of binary questions about safer sex acts: “In the last 30 days, have you had oral sex only? Sex while sober? Sex while using a lubricant? Sex while using a pre- or post-exposure prophylaxis? Sex restricted to one partner? Sexual abstinence?”
12. Proportion of participants in each group who reported engaging in one or more HIV preventive care-seeking or information-seeking acts in the last week (measured weekly in weeks 1 to 26): During the weekly text message survey, participants will be asked about HIV prevention care-seeking and information-seeking: “In the last 7 days, have you obtained an HIV test? Received for free or paid for condoms? Discussed HIV testing, condom use, or HIV medications with a sexual partner? Taken any HIV preventive medications?”
13. Proportion of participants in each group who reported engaging in one or more HIV preventive care-seeking or information-seeking acts in the last month (measured in weeks 1 and 26): During the in-person pre-intervention and post-intervention assessments, participants will be asked a list of binary questions about HIV prevention care-seeking and information-seeking: “In the last 30 days, have you obtained an HIV test? Received for free or paid for condoms? Discussed HIV testing, condom use, or HIV medications with a sexual partner? Taken any HIV preventive medications? Discussed prevention of HIV with a counselor or in an organized group session?”
14. Proportion of participants in each group who reported doing one or more paid hours of work in the last week (measured weekly in weeks 1 to 26): During the weekly text message survey, participants will be asked about paid hours of work: “In the last 7 days, how many hours did you work for pay?”
15. Proportion of participants in each group who reported doing one or more paid hours of work in the last month (measured in weeks 1 and 26): During the in-person pre-intervention and post-intervention assessments, participants will be asked about paid hours of work: “In the last 30 days, how many hours did you work for pay per week, on average?”

The relevance of the secondary outcomes is to examine the completion and level of employment and behavioral outcomes pertaining to efficacy assessment in a potential larger trial. Outcomes 8 and 9 will assess sexual risk behaviors. Outcomes 10, 11, 12, and 13 will assess HIV preventive behaviors. Outcomes 14 and 15 will assess employment. During the in-person post-intervention assessments, participants will also be asked six questions about the acceptability of the comparison and experimental interventions and the text messaging survey:

1. How much did you like the intervention?
2. How much help to you was the intervention in improving your ability to earn income?
3. How much help to you was the intervention in improving your ability to prevent HIV?
4. How likely are you to recommend the intervention to a friend?
5. How much did you like the text message survey?
6. How easy was it to respond each week to the text message survey?

All in-person assessments will be conducted in a private setting at the CBO centers using a structured questionnaire that will be orally administered by a trained research assistant. The questionnaire was developed for this study, and its validity and reliability are not known. The expected duration of each assessment is 30 min. To minimize under-reporting of negative outcomes, the financial incentives of the experimental group are not conditioned on self-reported behaviors. Rather, the financial incentives are conditioned only on attendance at the weekly educational sessions, responding to the weekly text message survey, and demonstrating use of the grant payments for business-related expenses.

### Additional process documentation

In addition to the feasibility outcomes described above, the PI or an SRC will use the structured weekly participant checklist to document whether each participant experienced an intervention-related adverse event. A second structured checklist, called the session checklist, will also be completed by the PI or an SRC over the course of each educational session to document the provision of key messages and activities. This will support documentation of the extent of delivery of the educational sessions. We will not audio-record the educational sessions to protect the privacy and confidentiality of participants in discussing sensitive financial and sexual behaviors.

### Statistical analysis

To examine feasibility, the analysis of this study will be descriptive as described in the outcome measures section (i.e., outcomes 1 to 7). Descriptive statistics will be reported for the primary and secondary outcomes for the specified time points for all participants and by study group. Frequencies and proportions will be used to summarize binary and categorical data. Means and standard deviations (or medians and interquartile ranges) will be used to summarize continuous data. Study randomization and retention will be described using a standard CONSORT diagram. We will report the number of enrolled participants, number and percentage of participants completing each of the run-in requirements, number and percentage of participants randomized to the comparison or experimental intervention, number and percentage of participants retained or withdrawn at the end of the interventions, and number and percentage of participants completing the post-intervention assessment. Participants must complete their in-person post-intervention assessment between weeks 26 to 30 to be treated as non-missing. The weekly text message survey will be sent every Friday. Participants must respond to the weekly text message survey by Monday to receive the payment for responding and to be treated as not missing.

Using the repeated longitudinal data from the weekly text message surveys, we will explore changes in sexual risk, HIV preventive behaviors, and employment using weekly outcomes 8, 10, 12, and 14. First, we will partition the study period into exposure periods: before the start of the interventions (weeks 1 to 3, pre-exposure), during the interventions (weeks 4 to 23, exposure), and following the cessation of the intervention (weeks 24 to 26, post-exposure). Second, we will explore differences in the level and trend of weekly employment, sexual risk, and HIV preventive behaviors between the comparison and experimental groups over time. A graphical summary of the weekly data will be presented. In addition, a random-effects generalized linear model will be used to explore the variability in the participants’ pre-exposure behaviors (random intercepts) and to explore the variability of participant trajectories in behaviors over the exposure period (random slopes). A logistic regression will be used for the dichotomous measures. Third, we will explore the level and trend in employment, sexual risk, and HIV preventive behaviors in the post-exposure period relative to the pre-exposure and exposure periods to explore sustainability.

We will use these data to inform the power calculation in a larger trial. If weekly text message surveys are found not to be a feasible method for measuring study outcomes over time, we will explore changes in sexual risk, HIV preventive behaviors, and employment using outcomes 9, 11, 13, and 15. All statistical analyses will be performed using STATA SE Version 15.1 or later (StataCorp LLC, College Station, TX). We will use both the intention-to-treat analysis sample (defined as every participant randomized) and the per-protocol analysis sample (defined as only participants completing 70% or more of the intervention activities). The results of the analysis will be used to guide the study design, selection of primary outcome(s), and sample size calculation of a future trial.

### Data management

Outcome data will be anonymized using a unique study identification number and stored separately on a password-protected computer. All hard copies of study assessment forms will be kept in a locked cabinet or drawer in the locked offices of the PI and SRCs. A hard copy of a record sheet linking participant identity, contact details, and study identification number for all participants will be kept separately by the PI and SRCs in a locked cabinet or drawer. To promote data quality, data ranges will be used to restrict invalid entries. All assessment data will be reviewed by the PI or a trained research assistant to ensure accuracy and completion.

### Data monitoring

To maintain confidentiality: (1) all study assessments will be conducted in a private setting, (2) all study staff will sign a confidentiality agreement to ensure they keep study information private, (3) participants will be told how they can maximize their privacy when responding to the text message survey (i.e., using a phone password, deleting responses, etc.), and (4) presentations and publications of study findings will use only aggregate data and will not identify individuals. To minimize the research burden on participants: (1) all study activities will be conducted during regular service hours at the participants’ affiliated CBO and (2) study assessments will collect the minimum amount of information needed to answer the study’s feasibility questions. Participants who experience psychological distress because of responding to questions perceived to be sensitive will be referred to support services at the CBO. There are no planned provisions for ancillary or post-trial care.

All study staff will be required to report adverse events that may be associated with receipt of the interventions and to report severe adverse events (such as death, impairment, disability, hospitalization, or any life-threatening event) to the PI. The PI will report all adverse events and severe adverse events through an adverse event report to the institutional review board of Johns Hopkins University School of Public Health within 48 h of receiving the notification or observing the event. A summary of the adverse events and severe adverse events that occurred during the year will be included in the annual progress report to the study’s funder. Because the interventions are associated with minimal risk to participants, a data monitoring committee that is independent from the sponsor was not deemed necessary.

### Criteria for progression to a larger trial

The decision of whether and how to proceed to a larger trial will be based on the feasibility data and overall study experience. For progression to a full-scale trial, we will consider the study’s two primary outcomes as criteria: (1) response of 70% or more of participants in both groups to 70% or more of the weekly text message surveys and (2) completion by 70% or more of the participants assigned to the experimental intervention of 70% or more of the experimental intervention activities, such as text message receipt, session attendance, grant spending, and mentor contact. We will also consider whether the study recruits at least 80% of the target sample and the acceptability of the comparison and experimental interventions. Failure to meet one or more criteria will be sufficient reason to consider modifying the interventions or the study design before conducting a larger trial.

### Dissemination policy

We intend to disseminate the outcomes of this feasibility trial in publications authored by the study team in peer-reviewed journals, the ClinicalTrials.gov registry, and relevant community partner meetings, scientific conferences, and colloquia. We will also share the results with our participants.

## Discussion

This feasibility trial will inform whether an efficacy randomized clinical trial is needed and how to conduct a larger trial. The feasibility design will enable us to examine the uncertainties that would arise when planning for a larger trial, such as participant willingness to be randomized, the time needed to collect data, the tolerability of the intervention, and the response rates to outcome assessments [59, 60]. Currently, there is limited research on community-based interventions in the U.S. that address economic disparities as they relate to HIV. The experimental intervention draws from prior published HIV prevention microenterprise interventions conducted primarily in low-income countries and targeting women [13, 44–52]. We have adapted these models for use in an impoverished U.S. urban setting with a focus on both African-American women and men. In addition, while text messages have been used in the implementation of other HIV risk reduction studies [61–64], text messages have not previously been combined with a microenterprise element and an intervention informed by behavioral economics, such as this one. The intervention aims to reduce sexual risk behaviors and increase employment and uptake of HIV preventive behaviors by building skills, and through motivational messaging and financial support. The comparison intervention includes job announcements only, which is comparable to the usual employment support provided at the participating community centers. The rationale for including HIV educational advice in the experimental intervention is to enable participants to translate financial empowerment into sexual health empowerment using new resources and skills to prevent HIV.

A limitation of this trial is the recruitment of economically-vulnerable young adults who are receiving supportive residential services (i.e., emergency shelter and transitional housing) at the study’s CBOs rather than recruiting more at-risk youth who are disconnected from supportive services. The trial is based within existing CBOs to promote retention, sustainability, and referrals to housing services. Therefore, the feasibility of implementing the study outside of existing community organizations will not be known. A strength of the trial is that it includes a relatively long and multi-faceted intervention that may plausibly have positive effects. It also builds on prior formative research conducted by the PI regarding participant interests and norms [40–42].

Should the intervention be feasible and safe, the study could be readily scaled up to a larger efficacy trial. If found to be effective, the EMERGE Project has the potential to make meaningful economic and health improvements.

### Trial status

Recruitment began in December 2018 and was completed in February 2019. The current protocol is version 5, dated 23 January 2019. The trial is currently ongoing.

## Additional file

Additional file 1: SPIRIT 2013 checklist: recommended items to address in a clinical trial protocol and related documents. (PDF 33600 kb)

## Data Availability

The de-identified dataset used to analyze the results from this study, along with the final protocol, will be available in an online repository.

## Abbreviations

CBO: Community-based organization
EMERGE: Engaging Microenterprise for Research Generation and health Empowerment
HIV: Human immunodeficiency virus
PI: Principal investigator
SRC: Senior research coordinator
